# Comparative Evaluation of Booster Vaccine Efficacy by Intracoelomic Injection and Immersion with a Whole-Cell Killed Vaccine against *Lactococcus petauri* Infection in Rainbow Trout (*Oncorhynchus mykiss*)

**DOI:** 10.3390/pathogens12050632

**Published:** 2023-04-22

**Authors:** Tryssa de Ruyter, Eric Littman, Zeinab Yazdi, Mark Adkison, Alvin Camus, Susan Yun, Timothy J. Welch, William R. Keleher, Esteban Soto

**Affiliations:** 1 Department of Medicine and Epidemiology, School of Veterinary Medicine, University of California–Davis, Davis, CA 95616, USA; 2 School of Veterinary Medicine, University of Pennsylvania, Philadelphia, PA 19104, USA; 3 California Department of Fish and Wildlife, Rancho Cordova, CA 95670, USA; 4 Department of Pathology, College of Veterinary Medicine, University of Georgia, Athens, GA 30602, USA; 5 National Center for Cool and Coldwater Aquaculture, Kearneysville, WV 25430, USA; 6 Kennebec River Biosciences, Richmond, ME 04357, USA

**Keywords:** aquaculture vaccine, booster, emergent, injection, immersion, lactococcosis

## Abstract

*Lactococcus petauri* is an important emergent bacterial pathogen of salmonids in the USA. The purpose of this study was to evaluate the protection conferred to rainbow trout (*Oncorhynchus mykiss*) against *L. petauri* by formalin-killed vaccines in immersion and injectable forms, as well as the enhanced protection afforded by booster vaccination. In the first challenge, fish were immunized via intracoelomic injection (IC) or immersion (Imm) routes alone. Approximately 418 degree days (Temperature in degree Celsius × days post-immunization) (dd) Imm, or 622 dd IC post-vaccination, fish were challenged via IC with wild-type *L. petauri.* In the second experiment, initial Imm vaccination was followed by booster vaccination via Imm or IC routes 273 dd post-immunization along with appropriate PBS controls. The various vaccination protocol efficacies were evaluated by challenging fish with *L. petauri* by cohabitation with diseased fish 399 dd post-booster administration. A relative percent survival (RPS) of 89.5% and 28% was recorded in the IC and Imm single immunization treatments, respectively. In the second study, an RPS of 97.5%, 10.2%, 2.6% and −10.1% plus approximately 0%, 50%, 20%, and 30% bacterial persistence was recorded in the Imm immunized + IC boosted, Imm immunized + mock IC boosted, Imm immunized + Imm boosted, and Imm immunized + mock Imm boosted treatments, respectively. Only the Imm immunized + IC injection boosted treatments provided significant protection when compared to unvaccinated and challenged treatments (*p* < 0.05). In conclusion, although both Imm and IC vaccines appear safe for trout, the inactivated Imm vaccines seem to provide only mild and temporary protection against lactococcosis; whereas IC immunized trout develop a significantly stronger protective response in both challenges.

## 1. Introduction

Lactococcosis is an emerging disease of significant concern for American aquaculture. In fish, lactococcosis often presents as hemorrhagic septicemia with high mortality. Fish display erratic swimming, lethargy, exophthalmia, anorexia, darkening of the skin, and hemorrhage of the skin, fins, gills, or eyes [1,2,3]. While historically attributed solely to the gram-positive lactic acid bacterium *Lactococcus garvieae,* recent investigations have revealed that lactococcosis can additionally be caused by the closely related species *L. petauri* and *L. formosensis. Lactococcus petauri* was first characterized in 2017 after being isolated from a facial abscess of a sugar glider (*Petaurus breviceps*) [4] and shares 99.9% 16S rRNA sequence identity with *L. garvieae* subsp. *garvieae* ATCC 49156^T^ [5].

Piscine lactococcosis is particularly prevalent in salmonids in water temperatures above 15 °C and causes significant economic losses in aquaculture through direct mortalities, as well as reduced production and growth, increased labor costs, and treatment expenditures [3,6]. Of further concern, *L. garvieae* and *L*. *petauri* are also considered emerging zoonotic pathogens [7]. While *L. garvieae* was first described in 1983 as *Streptococcus garvieae* and has since been reported in multiple continents affecting many species of fresh and saltwater fish, *L. petauri* remained unrecognized as a fish pathogen until 2020 [8]. Additionally, until recently, mortalities from piscine lactococcosis outbreaks in cultured fish in the United States have been limited [9]. In 2020, however, four distinct California aquaculture facilities in Southern California and the Eastern Sierra experienced lactococcosis outbreaks in cultured rainbow trout (*Oncorhynchus mykiss*) resulting in the euthanasia of > 3.2 million fish due to ineffective therapeutic options [10]. These 2020 outbreaks were initially presumed to be *L. garvieae*, but whole genome sequence analysis of the etiological agent identified the isolates as *L. petauri* [11]. *Lactococcus petauri* continues to be a concern for California aquaculture, as in April 2022, another outbreak in the Eastern Sierra region of California resulted in the euthanasia of 350,000 rainbow trout [12]. Retrospective genome sequencing has further found that a 2007 piscine lactococcosis outbreak in a Greek rainbow trout production facility was the result of an *L. petauri* infection [1]. It is likely that additional previously assumed *L. garvieae* outbreaks may have been misidentified and were caused by *L*. *petauri.*

While overwhelming evidence supports the use of vaccination to prevent disease losses in aquaculture, no licensed vaccine against piscine lactococcosis is currently available in the USA. Furthermore, antibiotic stewardship remains a priority, as inappropriate use can lead to resistance in fish and human bacterial pathogens. Despite the need, an effective vaccine against lactococcosis remains elusive, possibly resulting from the classification of multiple ambiguous taxa as *Lactococcus* spp. that are biochemically and phenotypically similar but genetically and immunogenically distinct. In response to these issues, inactivated autogenous vaccines manufactured using farm or region-specific bacterial strains are gaining acceptance in the US aquaculture industry. These vaccines can be deployed quickly since they are relatively simple to produce and are regulated less stringently than commercial/licensed vaccines, making their development and use comparatively inexpensive. These properties make custom autogenous vaccines an ideal tool for controlling and limiting the spread of rapidly emerging diseases [13]. Autogenous vaccines utilize inactivated whole-cell antigens and are typically applied via immersion (Imm) or intracoelomic (IC) injection routes in fish.

In immersion vaccines, the main target tissues are the mucosal surfaces of the skin, gills and nasal cavity, but antigenic uptake can occur in both the anterior and posterior gastrointestinal tract. Immersion vaccines are fairly efficacious and easy to administer. They can serve to vaccinate large numbers of small fish (1–5 g), and usually result in less stress to animals than injectable vaccines and display fewer side effects. However, they usually provide shorter-lasting protection with less systemic immune stimulation than injectable vaccines. This is particularly true if immersion baths are performed using killed organisms without an additional boost [14,15,16].

Parenteral (injectable) vaccines in fish typically induce a strong systemic immune response. However, injection requires fish to be handled individually, with the vaccine administered either manually or mechanically. In addition, injection site lesions are common. Although relatively uncomplicated in larger fish, vaccinating small fish by injection can be difficult and is not recommended for fish < 15 g in weight, complicating immunization in early life stages. In addition, the method is time-consuming and, if done manually, labor intensive. Additionally, at the immunological level, injectable vaccines are generally poor inducers of a mucosal response [14,15,16].

In this study, a representative *L. petauri* isolate from the 2020 California outbreak was used to evaluate the efficacy of an autogenous vaccine to prevent lactococcosis in a rainbow trout model of infection using both Imm and IC routes for immunization. Furthermore, primary Imm immunization followed by Imm and IC boost routes were also investigated to evaluate potential protocols that could be applied in aquaculture facilities with various life stages.

## 2. Materials and Methods

### 2.1. Fish

The experimental protocol and animals used were approved by the UC Davis Institutional Animal Care and Use Committee. Rainbow trout (*n* = 1200, ~2.4 g) were obtained from a local source with no history of lactococcosis. For verification, a sub-sample of the population (*n* = 20) was evaluated for underlying bacterial infection by complete clinical, bacteriological, and molecular analysis. Following clinical examination and fish necropsies, swabs of the posterior kidney and brain were inoculated onto Tryptic Soy Agar (TSA) with 5% sheep blood (SBA, Biological Media Service, UC Davis, Davis, CA, USA) for 96 h at 20 °C. Additionally, brain, spleen, and posterior kidney sub-samples (~50 mg each) were subjected to molecular analysis to ensure that they were free of *L. petauri* [10]. Fish were maintained in aerated 35-gallon tanks receiving 13–18 °C unchlorinated flow-through fresh water and fed a commercial trout feed (Skettring, Tooele, UT, USA) at 3% body weight per day. Dissolved oxygen levels were sustained at ~9 mg/L and monitored weekly. Water temperature was monitored daily. Fish were acclimatized for 1 month prior to immunization in the Center of Aquatic Biology and Aquaculture (CABA), Davis, CA, USA.

### 2.2. Bacteria and Vaccine Preparation

*Lactococcus petauri* JR1, originally isolated from cultured rainbow trout in Southern CA, which has been described previously by Shahin et al. 2021, was used in this study [10]. Bacteria were grown on SBA for 48 h at 28 °C. Formalin-killed vaccines were prepared by Kennebec River Bioscience in a USDA licensed autogenous vaccine facility. The inactivated bacteria were mixed with the adjuvant Montanide ISA^TM^ (Seppic, Cedex, La Garenne-Colombes, France) or Montanide IMS 1312 VG adjuvant for IC injection or Imm vaccination, respectively. Prior to immunization, feed was withheld for 24 h.

### 2.3. Single Intracoelomic Injection Vaccine Evaluation

Experimental treatments for fish immunized via the IC route consisted of immunized fish with a complete vaccine (≥1 × 10^8^ CFU formalin-killed bacteria/fish), sham-immunized with adjuvant alone (adjuvant), and positive and negative control groups that were sham-immunized with PBS. Each fish received an inoculum of 0.1 mL. Fish in the vaccine, adjuvant, and positive control groups were later challenged with *L. petauri* JR1. Negative control fish were handled similarly but injected with sterile PBS. Each treatment consisted of two replicate tanks with 25 fish per 35-gallon tank supplied with flow-through well water and constant aeration. All fish were withheld feed for 24 h prior to immunization.

Prior to injection or challenge, fish were anesthetized by immersion in sodium bicarbonate buffered tricaine methanesulfonate (MS-222, Syndel, Ferndale, WA, USA) at a dose of 50 mg/L before vaccination. All fish were maintained at 13 °C until 40 days post-immunization. Then, the temperature was increased by 1–2 °C per day in the system until a temperature of 17–18 °C was met.

Approximately 622 degree days (Temperature in degree Celsius × days post-immunization) (dd) post-injection vaccination, fish were anesthetized as previously described and challenged at 18 °C with 4.5 × 10^3^ CFU/fish wild type *L. petauri* JR1 via IC injection. Fish were monitored twice daily and any exhibiting moderate to severe clinical signs of disease (poor body condition, melanosis, hyperemia, loss of balance, lethargy, anorexia, scale protrusion and/or exophthalmia) were euthanized by an overdose of buffered Tricaine (MS-222; 250 mg/L) followed by complete clinical and bacteriological examinations. At the end of the challenge, five survivors per treatment were euthanized in buffered Tricaine (250 mg/L) and swabs from posterior kidneys were inoculated onto SBA agar to investigate bacterial persistence. The relative percent survival (RPS) was calculated according to the method described by Amend 1981: RPS = [1 − (% Mortality in vaccinated group/% mortality in control group)] × 100 [17].

### 2.4. Single Immersion Vaccine Evaluation

Evaluation of the efficacy of an Imm administered autogenous *L. petauri* vaccine using Montanide IMS 1312 VG adjuvant was performed using a similar experimental design and methodology as for the IC injection vaccine trial. Fish were relocated to an Imm bath and exposed for 30 s to the vaccine (≥10^9^ CFU formalin-killed bacteria/mL diluted 1:10 in source water before use), adjuvant alone, or PBS only (negative and positive controls) before being returned to their respective tanks. After a 23-day period during which water temperatures were increased from 13 °C to 17–18 °C, fish in the vaccine, adjuvant alone or positive control groups were challenged via IC injection with WT *L. petauri* 418-dd post-immunization. Negative control fish were exposed to PBS alone. All sampling was conducted as in the IC injection vaccine evaluation.

### 2.5. Efficacy of Intracoelomic and Immersion Boosting

Six experimental groups were used, which are summarized in Table 1. Treatment groups each consisted of five replicate tanks with 15 fish per 20-L tank supplied with flow-through well water (0.5 L per minute) and constant aeration. For initial immunization, treatment groups A-D were moved one tank at a time to an Imm bath of autogenous *L. petauri* vaccine following the manufacturer’s recommendations. Control groups E and F were treated with PBS only (Mock Immersion). All groups were immersed for 30 s before being returned to their respective tanks. Twenty-one days post initial immunization, treatment groups were booster vaccinated as per Table 1, following protocols similar to the above single Imm and IC immunization methods. Treatments boosted with a “Mock Immersion” were exposed to PBS only. Treatments boosted with a “Mock Intracoelomic” were injected with PBS only. Fish in the experimental groups were held at 13 °C for 15 days post-booster vaccination, after which temperatures were increased by 1–2 °C per day until a temperature of 17–18 °C was reached.

Twenty-eight days post-booster vaccination, groups A-E were exposed to *L. petauri* by cohabitation with five challenged trout/treatment tank. Serving as infectious “shedders”, naïve trout received 1000 CFU WT *L. petauri* JR1 by IC injection and had their adipose fins clipped for identification purposes. Morbidity and mortality were recorded twice daily for 21 days, noting shedder versus cohabitant fish. Clinically affected fish were euthanized by overdose of buffered MS-222 as previously described. Fish surviving the trial were euthanized and subjected to a complete necropsy. Posterior kidneys from 10 surviving treatment fish/tank were swabbed and inoculated onto SBA agar to evaluate bacterial persistence.

### 2.6. Statistical Analysis

Statistical analysis was performed in GraphPad^®^ Prism version 9.1.2 (GraphPad, San Diego, CA, USA). Survival curves were compared with Log-rank (Mantel-Cox) and Gehan-Breslow-Wilcoxon tests. In all cases, replicates were pooled as there was no significant difference between the curves at a 95% confidence level using the Log-rank (Mantel–Cox) test. A *p*-value of ≤0.05 was considered statistically significant for all tests.

## 3. Results

### 3.1. Protection Conferred by Single Intracoelomic Injection Vaccine

**Figure 1:**
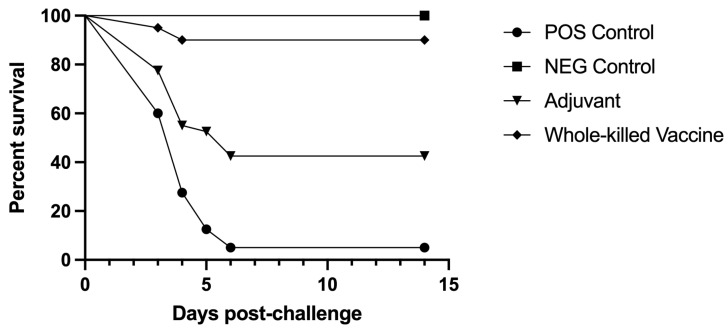
Percent survival of rainbow trout (*Oncorhynchus mykiss*) in all injectable treatment groups after IC injection challenge with *L. petauri.* Injectable vaccine and adjuvant only survival curves are significantly different from the positive control (*p* < 0.001). The adjuvant only group has significantly lower survival compared to the injectable vaccine group (*p* < 0.001). RPS for the injectable vaccine is 89.5% and RPS of adjuvant only is 39.5%.

The first mortalities for IC immunized fish occurred 3 days post *L. petauri* challenge (Figure 1). The injectable vaccine conferred significant protection against *L. petauri* induced lactococcosis when compared to the other treatments, resulting in an RPS of 89.5% (*p* < 0.001). Adjuvant only immunized fish had an RPS of 39.5% and presented significantly greater survival than the unvaccinated and challenged treatment group, but significantly lower survival than fish immunized by injection (*p* < 0.001). All moribund or dead fish yielded positive isolation of *L. petauri* regardless of treatment. *Lactococcus petauri* was recovered from 2/2 positive control survivors and 4/5 adjuvant immunized survivors. No bacteria were isolated from the injection vaccinated survivors or negative control fish.

### 3.2. Protection Conferred by Single Immersion Vaccination

**Figure 2:**
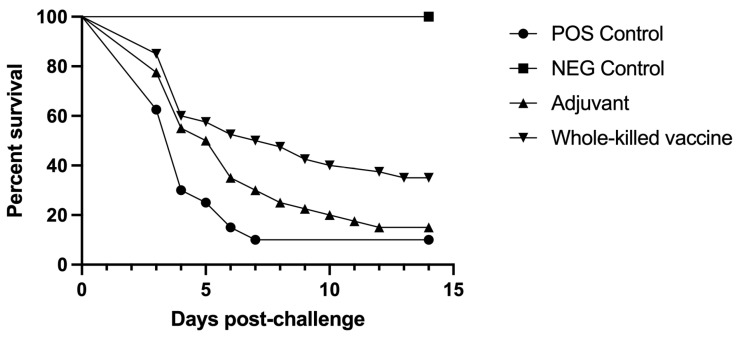
Percent survival of rainbow trout (*Oncorhynchus mykiss*) in all Imm treatment groups after IC injection challenge with *L. petauri*. Imm vaccination has a significantly higher survival curve compared to the positive control (*p* = 0.0006). The Imm vaccination group has an RPS of 28%. The adjuvant only group has a similar survival curve to the positive control.

The first mortalities for Imm immunized fish occurred three days post *L. petauri* challenge (Figure 2). The Imm vaccine conferred significant, although limited, protection against *L. petauri*, resulting in an RPS of 28%, but a significantly higher survival when compared to the positive control group (*p* = 0.0006). Adjuvant only immunization resulted in an RPS of 6%, similar survival to positive controls, and significantly lower survival when compared to Imm vaccine immunized fish (*p* < 0.04). *Lactococcus petauri* was isolated from all moribund or dead fish regardless of treatment as well as 1/4 unvaccinated and challenged survivors and 1/5 Imm vaccinated and challenged survivors.

### 3.3. Protection Conferred by Booster Vaccination and Cohabitation Challenge

**Figure 3:**
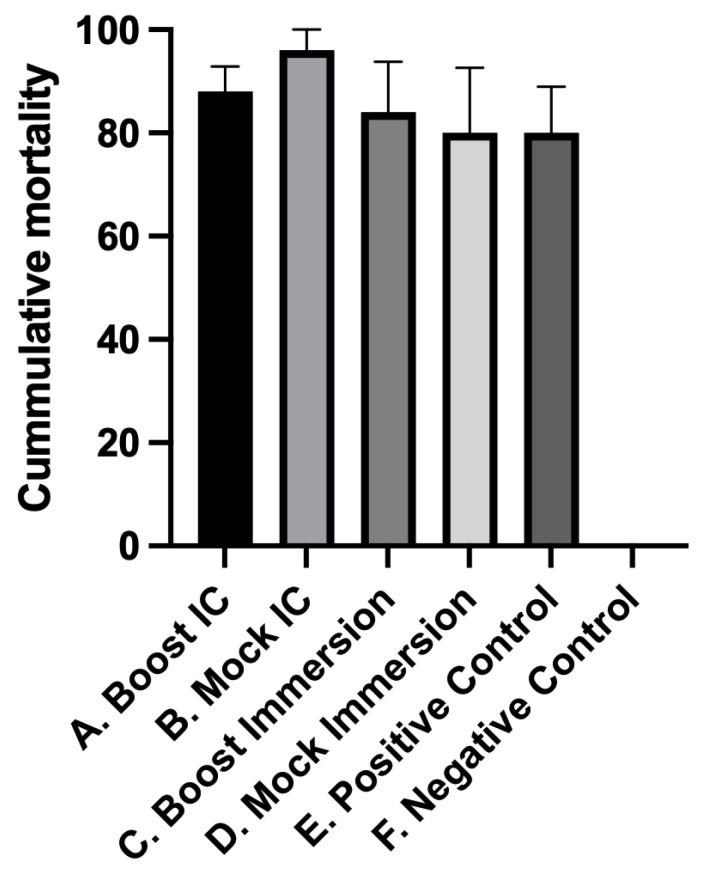
Cumulative mortalities of infected “shedder” rainbow trout (*Oncorhynchus mykiss*) 21d post-intracoelomic challenge with *L. petauri*. Negative Control (treatment F) is significantly different (*p* < 0.001) from all other treatments. Shedders in Positive Control (treatment E), Boost IC (treatment A), Mock IC (treatment B), Boost Imm (treatment C), and Mock Imm (treatment D) all had similar mortalities. Error bars represent standard errors for quintuplicate replicate tanks.

**Figure 4:**
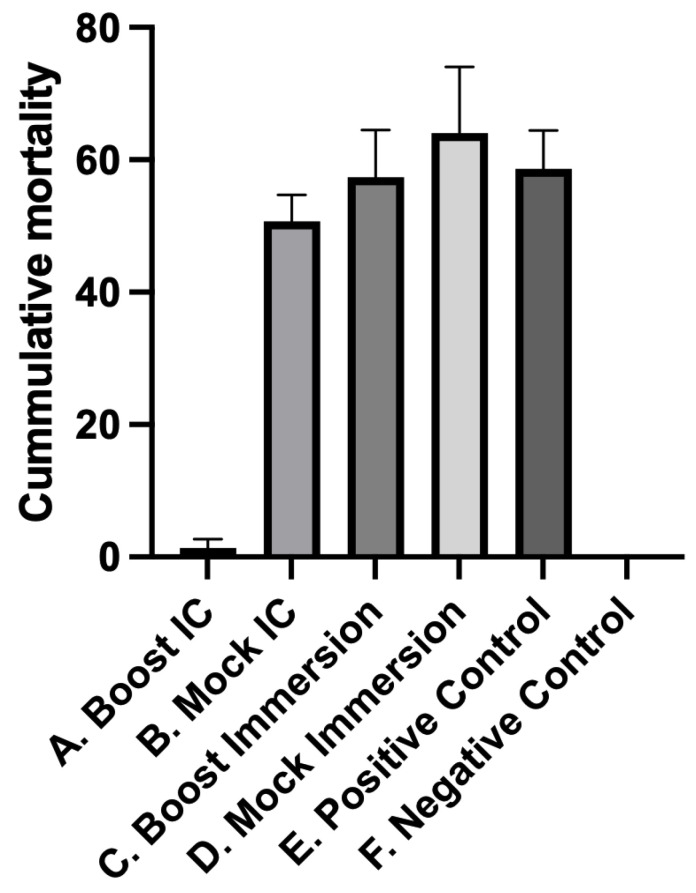
Cumulative mortalities of “cohabitant” rainbow trout (*Oncorhynchus mykiss*) 21 days post-cohabitation challenge with *L. petauri* infected shedders. Negative Control (treatment F) and Boost IC (treatment A) had significantly less mortality (*p* < 0.001) to all other treatments. Positive Control (treatment E), Mock IC (treatment B), Boost Imm (treatment C), and Mock Imm (treatment D) had similar mortalities to each other. Error bars represent standard errors for quintuplicate replicate tanks.

**Figure 5:**
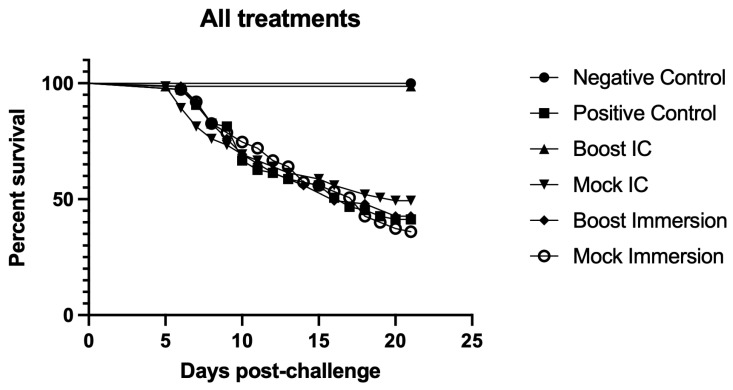
Percent survival of cohabitant rainbow trout in all treatment groups after challenge with *L. petauri*. Negative Control (treatment F) and Boost IC (treatment A) survival curves are significantly different (*p* < 0.001) from all other treatments. Positive Control (treatment E), Mock IC (treatment B), Boost Imm (treatment C), and Mock Imm (treatment D) have similar survival curves to each other.

In contrast to IC injection, which bypasses initial host colonization and innate protective barriers, the cohabitation study simulates natural disease transmission by introducing infected shedding individuals to promote pathogen uptake through regular portals of entry [18]. Cumulative mortalities among infected shedder fish were similar between treatment groups (Figure 3). Cumulative mortalities in the rainbow trout treatment groups following *L. petauri* challenge by cohabitation are shown in Figure 4. No mortality or clinical signs were observed in the unexposed negative control group. The first cohabitant mortalities occurred 5 days post-challenge in treatments A (Boost IC) and B (Mock IC). The first mortalities in the positive controls, treatment C (Boost Imm), and treatment D (Mock Imm) occurred 6 days post-challenge (Figure 5). Moribund and dead fish exhibited similar signs and pathologic changes indicative of septicemia, including erratic swimming, melanosis, exophthalmia, and internal hemorrhage. Additional changes included cloudy cerebrospinal and coelomic fluid suggestive of meningitis and coelomitis, respectively. Significant protection against *L. petauri* only occurred in the IC boostered fish (Figure 5), where an RPS of 97.5% was observed. The RPS and bacterial persistence results for all treatment groups are presented in Table 1.

## 4. Discussion

The results of the current study demonstrate that an autogenous formalin-inactivated vaccine confers significant protection against *L. petauri*. However, when assessed by greater RPS and lower bacterial persistence, the protection provided by IC injection is greater than by Imm (89.5 vs. 28%). Immunostimulation by Imm vaccination resulted in a moderate, but significant, increase in RPS when fish were challenged with *L. petauri* 418 dd post-immunization, suggesting further research on the potential for mucosal vaccines is warranted.

Mucosal vaccines, including the Imm vaccine used here, aim to stimulate strong immune responses at mucosal sites, such as the gut, gills, skin and nares to protect against infection by corresponding pathogens, and are highly desired by the global aquaculture industry due to their ease of administration [16]. However, antigen uptake across mucous membranes can be poor, resulting in lower systemic immune responses and shorter protection times compared to parenteral vaccines [19,20].

Other mucosal vaccine strategies, such as oral vaccines, are widely accepted by the aquaculture industry; however, inactivated oral vaccines against *L. garvieae* infections in trout failed to provide protection as strong as injectables in laboratory-controlled challenges in rainbow trout [21]. However, augmented approaches to oral vaccination—such as the use of polymer vehicles to coat antigens and prevent degradation, like the chitosan-alginate coated vaccines against *L. garvieae* and *S. iniae*—improve immunostimulation and protection in rainbow trout [19,22].

In contrast, whole cell killed bacterins injected via IC are highly effective against piscine lactococcosis in rainbow trout [21,23,24,25], Nile tilapia *Oreochromis niloticus* [26], grey mullet *Mugil cephalus* [27], and sorubim *Pseudoplatystoma* sp. [28] at preventing infection and controlling mortality. While typically producing strong systemic innate and adaptive immune responses, mucosal responses are generally poor. Injectable vaccines also require fish to be handled, which is time consuming, labor intensive, logistically difficult with small fish, and it potentially induces stress [16].

Following primary immunization, booster vaccination is used to increase or extend immune protection, although it may not be feasible for some aquaculture sectors where production cycles are short or other logistical issues exist. However, oral booster vaccination has been adopted by some European aquaculture producers to combat piscine lactococcosis [29], and in combination with supplements like alginate microparticles have resulted in >85% RPS in some studies following initial IC vaccination [21]. The IC injection vaccine we present here, with an RPS of 89.5%, is similar in efficacy to the other single intraperitoneal injection vaccine against lactococcosis in rainbow trout in the literature, with an RPS of 94% [24]. The chitosan-alginate coated vaccine against lactococcosis in rainbow trout fed for 14 days had a slightly lower RPS of 72.18 ± 9.8% [22]. The Imm primary and IC injection booster vaccination combination we present here conferred the highest protection against lactococcosis in rainbow trout of all other methods with an RPS of 97.5%.

In the current cohabitation transmission study, the Imm primary and IC injection booster vaccination combination conferred protection against *L. petauri*, producing an RPS of 97.5%. Primary Imm vaccination followed by an Imm booster produced a RPS of only 2.6%, indicating protection by Imm vaccination alone is weak and short-lived. Alternatively, the use of an Imm booster could have induced “tolerance” at mucosal and/or systemic sites, resulting in poor immunoprotective response. Tolerance following mucosal immunization has been previously reported when using inactivated vaccines, and further research is needed to provide better options for the immunization of early life stages fish where parenteral vaccines are logistically or economically non-feasible [30,31].

In conclusion, this study demonstrates that single IC injection and Imm vaccines produce significant levels of protection against *L. petauri* in laboratory challenges, although vaccination by IC injection vaccine was far more effective at protecting rainbow trout against *L. petauri* than the Imm vaccine, with RPS values of 89.5 vs. 28%. In cohabitation trials with infected trout, the combination of primary Imm and IC injection booster vaccination produced the greatest survival (97.5%). Results demonstrate the potential for the use of autogenous vaccines to protect against losses from *L. petauri* induced lactococcosis in rainbow trout, and represent a start for the development of an effective immunization methodology. However, further research is needed to elucidate an “ideal” vaccination scheme amenable to adoption by the trout industry that results in safe, economical, and logistically sound vaccination protocols.

## Figures and Tables

**Table 1:** Immunization protocols and the overall efficacy of different treatments used to boost rainbow trout (*Oncorhynchus mykiss*) against piscine lactococcosis.

Treatment	Initial Vaccine	Boost Vaccine	Challenge	RPS	Bacterial Persistence
A	Immersion	Intracoelomic	Cohabitant	97.5%	0 out of 10
B	Immersion	Mock Intracoelomic	Cohabitant	10.2%	5 out of 10
C	Immersion	Immersion	Cohabitant	2.6%	2 out of 10
D	Immersion	Mock Immersion	Cohabitant	−10.1%	3 out of 10
E	Mock Immersion	Mock Immersion	Cohabitant	--	3 out of 10
F	Mock Immersion	Mock Immersion	Non-exposed	--	--

## Data Availability

All data generated or analyzed during this study are included in this published article.
